# Conformations of tissue plasminogen activator (tPA) orchestrate neuronal survival by a crosstalk between EGFR and NMDAR

**DOI:** 10.1038/cddis.2015.296

**Published:** 2015-10-15

**Authors:** T Bertrand, F Lesept, A Chevilley, S Lenoir, M Aimable, A Briens, Y Hommet, I Bardou, J Parcq, D Vivien

**Affiliations:** 1 Inserm, Inserm U919, Serine Proteases and Pathophysiology of the Neurovascular Unit, University Caen Basse-Normandie, GIP Cyceron, Caen, France

## Abstract

Tissue-type plasminogen activator (tPA) is a pleiotropic serine protease of the central nervous system (CNS) with reported neurotrophic and neurotoxic functions. Produced and released under its single chain form (sc), the sc-tPA can be cleaved by plasmin or kallikrein in a two chain form, tc-tPA. Although both sc-tPA and tc-tPA display a similar fibrinolytic activity, we postulated here that these two conformations of tPA (sc-tPA and tc-tPA) could differentially control the effects of tPA on neuronal survival. Using primary cultures of mouse cortical neurons, our present study reveals that sc-tPA is the only one capable to promote *N*-methyl-D-aspartate receptor (NMDAR)-induced calcium influx and subsequent excitotoxicity. In contrast, both sc-tPA and tc-tPA are capable to activate epidermal growth factor receptors (EGFRs), a mechanism mediating the antiapoptotic effects of tPA. Interestingly, we revealed a tPA dependent crosstalk between EGFR and NMDAR in which a tPA-dependent activation of EGFRs leads to downregulation of NMDAR signaling and to subsequent neurotrophic effects.

Tissue-type plasminogen activator (tPA) is secreted by endothelial cells and promotes fibrinolysis via the conversion of fibrin-bound plasminogen into plasmin.^1^ Neurons and some glial cells also secrete tPA.^2, 3, 4, 5^ tPA is secreted as a single-chain form (sc-tPA), which can be processed into a two-chain form (tc-tPA) by plasmin or kallikreins.^6, 7^ Interestingly, sc-tPA is proteolytically active even without proteolytic processing. In addition to its vascular functions, tPA displays critical roles in the brain parenchyma with roles in cell migration, neuronal plasticity and survival,^8, 9, 10, 11, 12, 13, 14^ acting either as an enzyme or as a cytokine-like molecule. Among its actions, tPA is well described to promote neurotoxicity, likely through promotion of *N*-methyl-D-aspartate receptor (NMDAR) activity.^15, 16, 17^ Recently, we reported that only sc-tPA can promote NMDAR signaling and neurotoxicity.^18^ Interestingly, data from wild-type mice,^19^ transgenic mice overexpressing tPA in neurons^20^ or *in vitro*^21^ also report neuroprotective effects of tPA.^9, 10^ The proposed mechanisms involved a tPA-dependent and non-proteolytic activation of either epidermal growth factor receptors (EGFRs)^22^ on oligodendrocytes or NMDARs.^20^

Here we explored a link between tPA conformations (sc-tPA and tc-tPA), EGFR- and NMDAR-dependent signaling pathways. Our findings identify sc-tPA as a selective positive modulator of NMDAR signaling in neurons when present at high concentrations and both sc-tPA and tc-tPA as positive modulators of EGFR signaling, this even at low concentrations. We also reveal a crosstalk between these two families of receptors, with the tPA-dependent activation of EGFRs reducing NMDAR signaling. By these mechanisms, sc-tPA and tc-tPA control neuronal death and survival.

## Results

### sc-tPA promotes and tc-tPA inhibits NMDAR signaling

**Figure 1:**
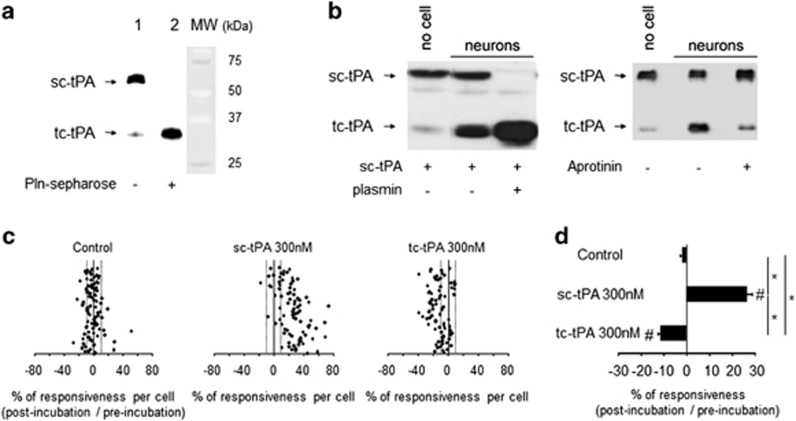
sc-tPA and tc-tPA differentially influence NMDAR signaling. (**a**) Sodium dodecyl sulfate-polyacrylamide gel electrophoresis (SDS-PAGE) followed by immunoblotting of sc-tPA and tc-tPA prepared as described in the Materials and Methods section (100 ng per lane). (**b**) SDS-PAGE followed by immunoblotting of sc-tPA and tc-tPA. sc-tPA was added on cultured cortical neurons 13 DIV for 1 h either alone or in the presence of plasmin or aprotinin. (**c**) Calcium video imaging performed on primary cultures of cortical neurons (12 DIV). After control NMDA stimulations (2 × 25 *μ*M, 30 s) used as baseline, neurons were incubated for 45 min in the presence of buffer (control, *n*=90 cells), sc-tPA or tc-tPA at 300 nM (sc-tPA, *n*=85 cells; tc-tPA, *n*=78 cells) prior to a second set of NMDA stimulations (2 × 25 *μ*M, 30 s). Percentages of potentiation or inhibition after incubation are calculated for each cell. (**d**) Percentage of potentiation or inhibition after incubation for each group (mean±S.E.M.; *P<0.0001 Kruskal–Wallis test followed by Mann–Whitney test; ^#^*P*<0.0001 Wilcoxon test comparison of preincubation and postincubation responses)

We first investigated NMDAR-induced calcium influx in the presence of either sc-tPA or tc-tPA on primary cultures of cortical neurons (Figure 1). tc-tPA was produced from human recombinant purified sc-tPA (Actilyse, see Materials and Methods, Figure 1a). sc-tPA and tc-tPA were characterized and used at equimolarity, as previously described.^18^ When added on neurons, sc-tPA is rapidly converted into tc-tPA (1 h), an effect increased by plasmin (5 nM). Moreover, the conversion of sc-tPA into tc-tPA is blocked by aprotinin (1 *μ*M), an inhibitor of plasmin (Figure 1b). sc-tPA (300 nM) promoted NMDA-induced neuronal calcium influx (80% of cells potentiated; 27% of potentiaton; *P<0.0001). On the contrary, tc-tPA (300 nM) led to a significant decrease in NMDA-induced calcium influx (51% of cells inhibited; 11% of inhibition for tc-tPA 300 nM; *P<0.0001; Figures 1c and d).

### Both sc-tPA and tc-tPA can promote EGFR signaling

**Figure 2:**
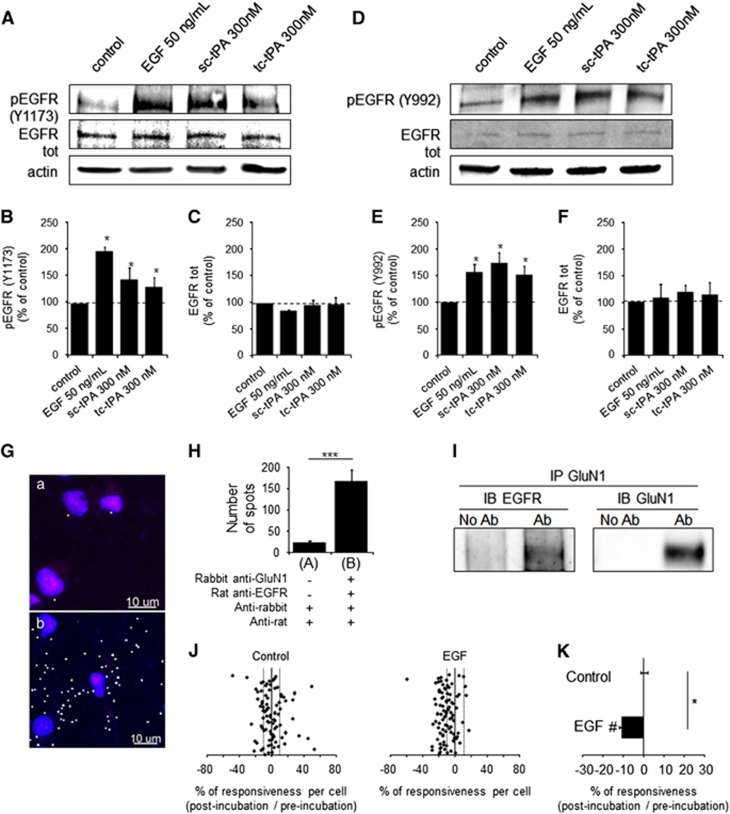
Both sc-tPA and tc-tPA at 300 nM can modulate EGFR signaling. (**a**) Representative immunoblots for phospho-EGFR (**A**–**C**: tyrosine 1173; **D**–**F**: tyrosine 992) and total EGFR on neurons (12–13 DIV) after treatments with EGF (50 ng/ml), sc-tPA or tc-tPA (300 nM) during 15 min. (**B**, **C**, **E** and **F**) Quantifications of phosphorylated EGFRs and total EGFRs were compared with control (mean±S.E.M.; *N*=3 or 4 experiments; *P<0.05). (**g**) Confocal images of endogenous NMDAR–EGFR complexes in cortical neurons. (**H**) Quantification of NMDAR–EGFR complexes detected by Proximity Ligation Assay (PLA). ***P-value<0.001; Mann–Whitney *U*-test, *N*=3. (**I**) Cross-immunoprecipitation assays demonstrating that EGFRs and NMDARs form stable complexes in cultured neurons. (**J**) After two NMDA stimulations used as baseline, neurons were incubated for 45 min with buffer (control, *n*=85 cells) or EGF 50 ng/ml (EGF, *n*=93 cells) prior to a second set of NMDA stimulations. Percentages of potentiation or inhibition after incubation are calculated for each cell. (**K**) Percentage of potentiation or inhibition after incubation for each group (mean±S.E.M.; *P<0.0001 Kruskal–Wallis test followed by Mann–Whitney *U*-test; ^#^*P*<0.0001 Wilcoxon test comparison of preincubation and postincubation responses)

Immunoblottings for phosphorylated EGFRs (phospho-tyrosines 1173 and 992) revealed that, similar to EGF (50 ng/ml), both sc-tPA and tc-tPA (300 nM) can activate neuronal EGFRs (Y1173: Figures 2A–C; +82%, +47%, +34% for EGF, sc-tPA and tc-tPA *versus* control, respectively; *P<0.05 and Y992: Figures 2D–F; +56%, +74%, +52% for EGF 50 ng/ml, sc-tPA and tc-tPA *versus* control, respectively; *P< 0.05). Proximity ligation assays (Figures 2G and H) and immunoprecipitation–immunoblotting assays (Figure 2I) revealed that NMDARs (NMDA receptor subunit 1 (GluN1) subunit) form complexes with EGFRs. Interestingly, EGF led to a reduction of NMDA-induced neuronal calcium influx (57% of cells inhibited; 11% of inhibition for EGF; *P<0.0001 when compared with controls; Figures 2J and K).

### tPA-dependent crosstalk between NMDARs and EGFRs

**Figure 3:**
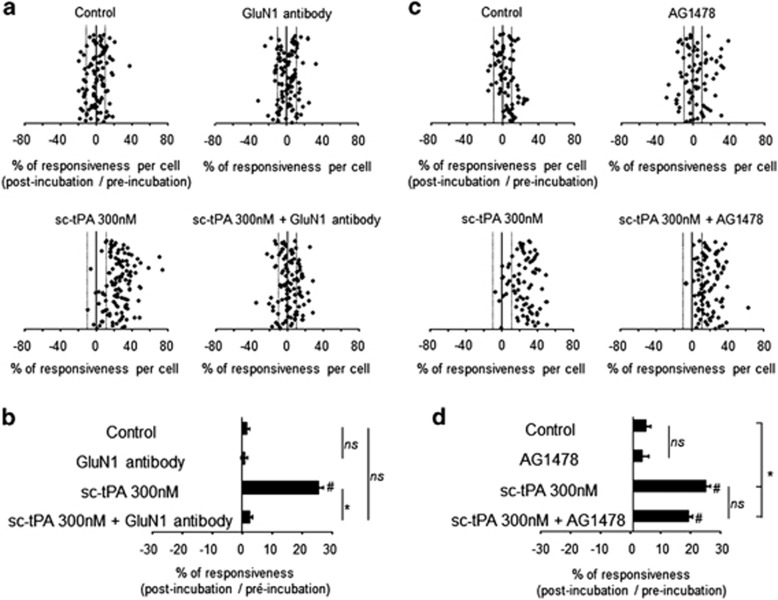
sc-tPA-promoted neuronal calcium influx is dependent on its interaction with NMDARs. Calcium video imaging performed on cortical neurons. (**a**) After two control NMDA stimulations used as baseline, neurons were incubated for 45 min in the presence of either buffer (control, *n*=104 cells), sc-tPA at 300 nM (sc-tPA *n*=111 cells) or GluN1 antibody at 10 *μ*g/ml (*n*=99 cells) alone or in combination (sc-tPA+GluN1 antibody; *n*=109 cells) prior to a second set of NMDA stimulations. Percentages of potentiation or inhibition after treatment are calculated for each cell. (**b**) Percentages of potentiation or inhibition after treatment are calculated for each cell and reported as percentages of responsiveness for each group. (**c**) In the same protocol, neurons were incubated for 45 min in the presence of buffer (control, *n*=75 cells), sc-tPA at 300 nM (sc-tPA, *n*=77 cells) or AG1478 at 5 *μ*M (AG1478, *n*=77 cells) alone or in combination (sc-tPA+AG1478, *n*=90 cells) prior to a second set of NMDA stimulations (2 × 25 *μ*M, 30 s). (**d**) Percentages of responsiveness for each group (mean±S.E.M.; *P<0.0001 Kruskal–Wallis test followed by Mann–Whitney test; ^#^*P*<0.0001 Wilcoxon test comparison of preincubation and postincubation responses). NS: not significant

**Figure 4:**
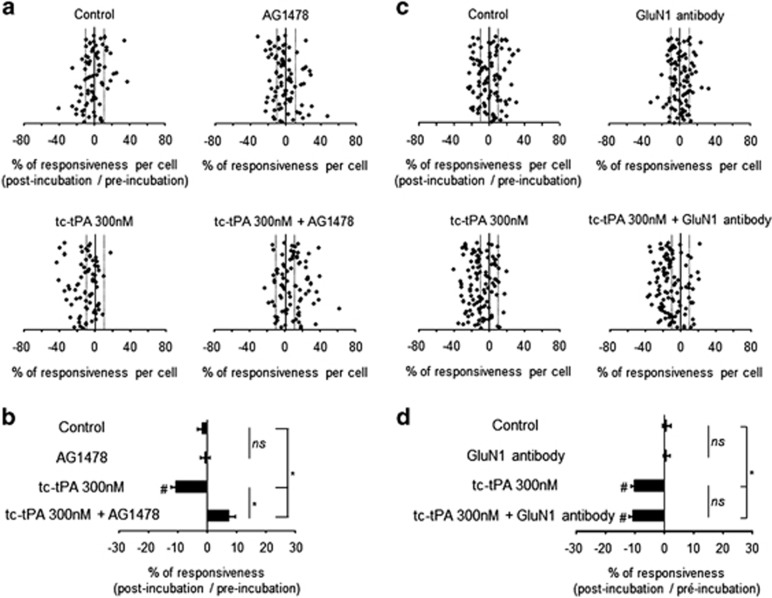
AG1478 reverses the inhibitory effect of tc-tPA on NMDAR signaling independently of its interaction with NMDARs. (**a**) After two NMDA stimulations used as baseline, neurons were incubated for 45 min in the presence of buffer (control, *n*=74 cells), AG1478 at 5 *μ*M (AG1478, *n*=84 cells) or tc-tPA at 300 nM alone or in combination (tc-tPA, *n*=70 cells; tc-tPA+AG1478, *n*=81 cells) prior to a second set of NMDA stimulations. Percentages of potentiation or inhibition after incubation are calculated for each cell. (**b**) Percentages of potentiation or inhibition after incubation are calculated for each individual cell and reported as percentages of responsiveness for each group. (**c**) In the same protocol, neurons were incubated for 45 min in the presence of buffer (control, *n*=99 cells), GluN1 antibody at 10 *μ*g/ml (GluN1 antibody, *n*=99 cells) or tc-tPA at 300 nM either alone or in combination tc-tPA 300 nM *n*=103 cells, tc-tPA+GluN1 antibody *n*=106 cells) prior to a second set of NMDA stimulations (2 × 25 *μ*M, 30 s). (**d**) Percentages of potentiation or inhibition after incubation are calculated for each individual cell and reported as percentages of responsiveness for each group (mean±S.E.M.; *P<0.0001, Kruskal–Wallis test followed by Mann–Whitney test; ^#^*P*<0.0001 Wilcoxon test of comparison of preincubation and postincubation responses). NS: not significant

NMDAR-induced calcium influx was studied in the presence of sc-tPA and tc-tPA (300 nM) alone or in the presence of either an inhibitor of the transphosphorylation of EGFRs (AG1478) or a GluN1 antibody previously characterized to prevent tPA-induced potentiation of NMDAR signaling^23^ (Figures 3 and 4). The potentiating effect of sc-tPA on NMDA-induced calcium influx (85% of cells potentiated; 25% of potentiation for sc-tPA 300 nM; *P<0.0001 when compared with controls) was completely prevented by GluN1 antibody (NS when compared with controls; *P<0.0001 when compared with sc-tPA alone; Figure 3a–c). In contrast, the presence of AG1478 failed to influence tPA-promoted NMDAR signaling (77% of cells potentiated; 19% of potentiation for sc-tPA 300 nM+AG1478; *P<0.0001 when compared with controls and NS: not significant when compared with sc-tPA alone, Figure 3d–f). In parallel, blockage of the trans-activation of EGFRs by AG1478 (Figures 4a–c) prevented tc-tPA-dependent inhibition of NMDAR signaling (*P<0.0001 considering the percentage of responsiveness for tc-tPA 300 nM alone *versus* tc-tPA 300 nM+AG1478; 46% *versus* 15% of cells inhibited in tc-tPA 300 nM and tc-tPA 300 nM+AG1478, respectively). tc-tPA-induced inhibition of NMDAR-mediated calcium influx was not modulated by the co-application of the GluN1 antibody (54% of cells inhibited; 10% of inhibition for tc-tPA 300 nM alone compared with 59% of cells inhibited and 11% of inhibition for tc-tPA 300 nM+GluN1 antibody; *P<0.0001; Figures 4d–f). Parallel experiments performed using another inhibitor of the activation of EGFR, Gefitinib (5 *μ*M), provided the same results as observed in the presence of AG1478 (Supplementary Figure 2). Altogether, these data reveal a crosstalk between NMDAR signaling and EGFRs in which tPA-mediated EGFR activation leads to an inhibition of NMDAR signaling.

### In a paradigm of NMDA-mediated excitotoxicity, only the sc-tPA promotes neuronal death

**Figure 5:**
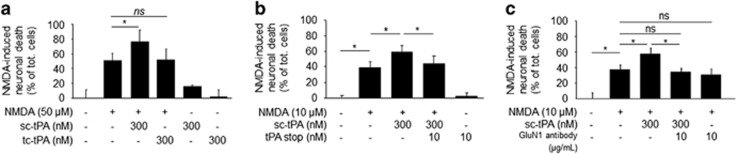
Only high concentrations of active sc-tPA promote excitotoxicity. (**a**) Cortical neurons (12-13 DIV) were exposed for 1 h to NMDA (50 *μ*M) in the presence of sc-tPA or tc-tPA. Neuronal death was quantified 24 h later. (**b** and **c**) Same experiments as in panel (**a**) were performed with neurons exposed for 24 h to NMDA (10 *μ*M) in the presence of sc-tPA alone or in combination with tPA stop (10 nM, **b**) or GluN1 antibody (10 *μ*g/ml, **c**). (mean±S.E.M.; *n*=3 experiments; 4 wells per condition; *P<0.05, NS: not significant, Kruskal–Wallis test followed by Mann–Whitney test)

NMDA-mediated excitotoxicity was tested on primary cultures of cortical neurons subjected to NMDA exposure in the presence of sc-tPA or tc-tPA (300 nM). As expected, NMDA-induced excitotoxicity was potentiated only in the presence of sc-tPA (+47% *P<0.05; Figure 5a). Co-application of tPA stop, an inhibitor of the proteolytic activity of tPA^24^ or the GluN1 antibody (see Figure 3) prevented the pro-excitotoxic effect of sc-tPA (*P<0.05; Figures 5b and c).

### In a paradigm of serum deprivation (SD)-induced apoptosis, both sc-tPA and tc-tPA are neuroprotective, an effect dependent on EGFR signaling

**Figure 6:**
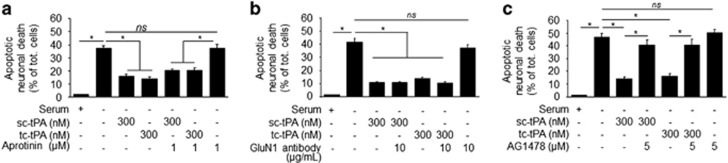
Both sc-tPA and tc-tPA rescue neurons from serum deprivation-induced apoptosis. (**a**) Neuronal death measured after a 24-h exposure to serum deprivation (SD) alone or in the presence of sc-tPA or tc-tPA at 300 nM alone or plus aprotinin (1 *μ*M; mean±S.E.M.; *n*=3 experiments; *P<0.05). (**b** and **c**) Neuronal death measured after a 24-h exposure to SD alone or in the presence of sc-tPA or tc-tPA plus GluN1 antibody (10 *μ*g/ml; **b**) or AG1478 (5 *μ*M; **c**) (mean±S.E.M.; *n*=3 experiments; 4 wells per condition; *P<0.05, NS: not significant, Kruskal–Wallis test followed by Mann–Whitney test)

In a paradigm of apoptotic neuronal death induced by deprivation of trophic factors in cortical neurons, both sc-tPA and tc-tPA displayed antiapoptotic properties. Aprotinin failed to prevent the antiapoptotic effect of sc-tPA, suggesting that the antiapoptotic effect of sc-tPA is not due to its previous conversion into tc-tPA (*P<0.05; Figure 6a). Blockage of the ability of tPA to promote NMDAR-induced calcium influx with the GluN1 antibody did not prevent the antiapoptotic effects of both sc-tPA and tc-tPA (*P<0.05, Figure 6b). However, blockage of the tPA-dependent transphosphorylation of EGFRs (AG1478) prevented the antiapoptotic activities of both sc-tPA and tc-tPA (*P<0.05, Figure 6c).

### Low concentrations of sc-tPA and tc-tPA are neurotrophic, an effect mediated by a crosstalk between EGFRs and NMDARs

**Figure 7:**
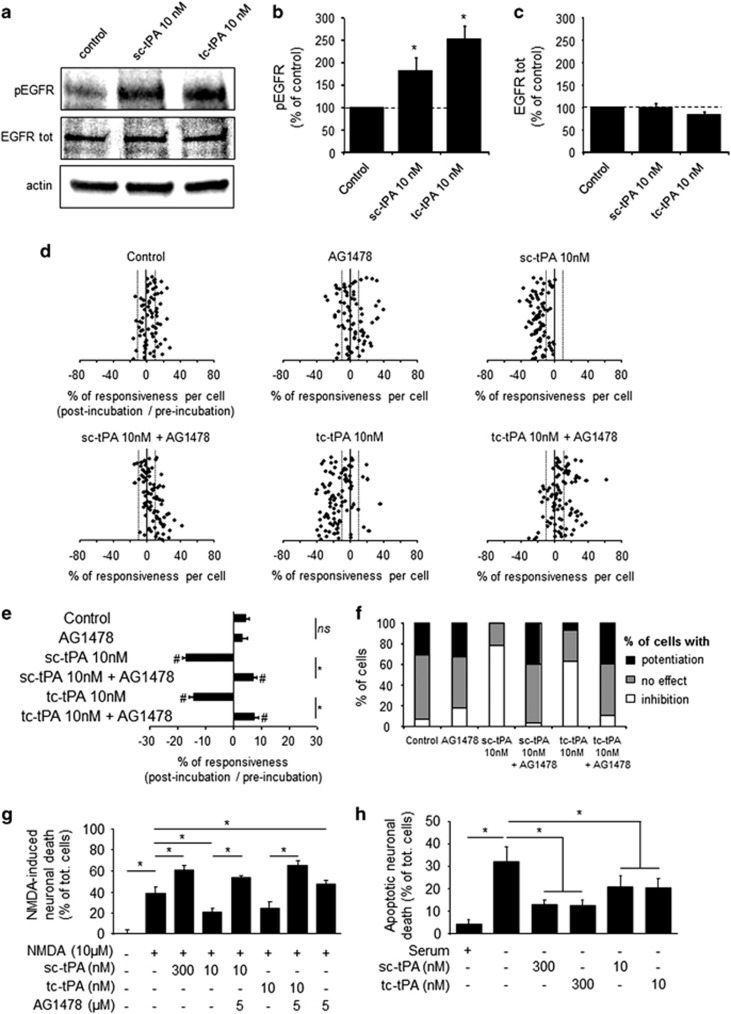
Both sc-tPA and tc-tPA at 10 nM promote EGFR signaling and are neuroprotective. (**a**) Representative immunoblots for phospho-EGFR (tyrosine 1173) and total EGFR on neurons after treatments with sc-tPA and tc-tPA (10 nM) during 15 min. (**b** and **c**) Quantification of phosphorylated EGFRs and total EGFRs compared with the control condition (*n*=3 experiments; *P<0.05). (**d**) After two NMDA stimulations used as baseline, neurons were incubated for 45 min in the presence of buffer (control, *n*=75 cells), AG1478 at 5 *μ*M (*n*=77 cells), sc-tPA (10 nM) alone or in combination with AG1478 (sc-tPA, *n*=78 cells; sc-tPA+AG1478, *n*=83 cells) or tc-tPA (10 nM) alone or in combination with AG1478 (tc-tPA, *n*=92 cells; tc-tPA+AG1478, *n*=92 cells) prior to a second set of NMDA stimulations. Percentages of potentiation or inhibition after incubation are calculated for each cell. (**e**) Percentages of responsiveness for each group (mean±S.E.M.; *P<0.0001 Kruskal–Wallis test followed by Mann–Whitney test; ^#^*P*<0.0001 Wilcoxon test comparison of preincubation and postincubation responses). (**f**) Percentages of cells either potentiated, inhbited or without effect for each group. (**g**) Cortical neurons were subjected to 24-h exposure to NMDA (10 *μ*M) in the presence of either sc-tPA or tc-tPA (10 nM) alone or in combination with AG1478 (5 *μ*M; *n*=3 experiments; 4 wells per condition; *P<0.05, NS: not significant; Kruskal–Wallis test followed by Mann–Whitney test). (**h**) Neuronal death measured after a 24-h exposure to either serum deprivation (SD) alone or in the presence of either sc-tPA or tc-tPA at 300 or 10 nM (*n*=3 experiments; 4 wells per condition experiments; *P<0.05, NS: not significant; Kruskal–Wallis test followed by Mann–Whitney test, mean±S.E.M.)

As it was previously reported that low concentrations of tPA may have protective effects through activation of NMDARs,^20^ we tested lower concentrations of sc-tPA and tc-tPA (10 nM; Figure 7) in our different paradigms. Immunoblotting for phosphorylated EGFRs revealed a sc-tPA- and tc-tPA-dependent (10 nM) activation of the EGFRs (+82 and +154% of activation for sc-tPA and tc-tPA at 10 nM, respectively; *P< 0.05; Figures 7a–c). Parallel experiments using calcium video microscopy revealed that sc-tPA and tc-tPA (10 nM) led to an inhibition of NMDAR signaling (78 and 63% of cells inhibited; 17 and 14% of inhibition for sc-tPA and tc-tPA (10 nM), respectively; *P<0.0001). This inhibitory effect was blocked by the co-application of AG1478 (3 and 11% of cells inhibited; 7 and 8% of potentiation for sc-tPA and tc-tPA at 10 nM+AG1478, respectively; *P<0.0001; Figures 7d–f). As an additional control, tc-tPA at 1 nM did not influence NMDA-induced calcium influx (Supplementary Figure 1).

Although sc-tPA at 300 nM promoted NMDAR-mediated excitotoxicity, 10 nM of either sc-tPA or tc-tPA protected neurons in the same paradigm of NMDAR-mediated excitotoxicity (−65% of excitotoxic death for sc-tPA, −60% for tc-tPA 10 nM; *P< 0.05, Figure 7g). Interestingly, blockage of the transphorylation of EGFRs (AG1478; Figure 7g) reversed these neuroprotective actions of sc-tPA and tc-tPA. As expected, both sc-tPA and tc-tPA displayed antiapoptotic properties even at low concentrations (−59% of apoptotic death at 300 nM and −35% at 10 nM; *P<0.05; Figure 7h).

**Figure 8:**
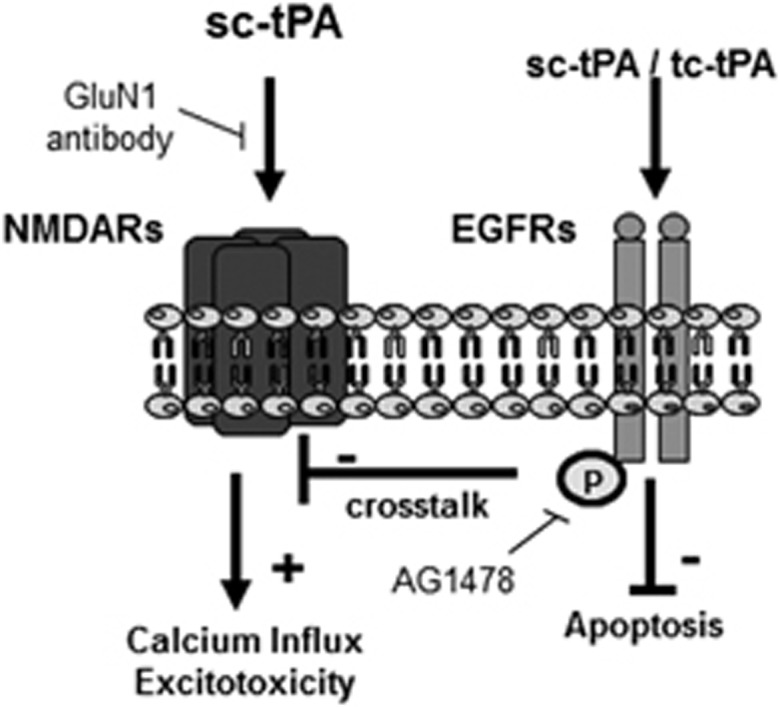
tPA-dependent crosstalk between EGFRs and NMDARs. sc-tPA-induced potentiation of NMDAR signaling and subsequent neurotoxicity, inhibited by GluN1 antibody. tPA also promotes EGFR signaling and subsequent antiapoptotic effects, independently of its conformation (sc-tPA and tc-tPA), an effect occurring even at low concentrations (down to 10 nM) and inhibited by AG-1478, an inhibitor of EGFR transphosphorylation. tPA-dependent activation of EGFRs leads to downregulation of NMDAR signaling and subsequent neurotrophic effects

Altogether, these data demonstrate that although direct activation of NMDARs by sc-tPA at high concentrations led to a pro-excitotoxic effect dependent of its proteolytic activity, lower concentrations of both sc-tPA and tc-tPA are antiexcitotoxic by a mechanism involving an EGFR-dependent downregulation of NMDAR signaling independently of their proteolytic activity. Our data also evidence that both sc-tPA and tc-tPA display antiapoptotic functions through a mechanism involving a direct activation of EGFRs and this independently of NMDARs (Figure 8).

## Discussion

We propose here a new scheme of the mechanisms through which tPA controls neuronal survival. We show that both conformations (sc-tPA and tc-tPA) have a neurotrophic effect by the activation of EGFRs. EGFRs can complex to NMDARs at the neuronal surface, orchestrating an original tPA-dependent crosstalk between both receptors, leading to a downregulation of NMDAR signaling and subsequent neurotrophic effects. However, when present at high concentration (300 nM), the sc-tPA promotes NMDAR signaling leading to an increased neuronal death, hiding the neurotrophic effects of lower concentrations of tPA.

tPA-driven control of neuronal fate could also depend on the different subtypes of NMDAR subunits involved, as well as on the location of the receptors (synaptic *versus* extrasynaptic). Specifically, exogenous tPA can not only promote neurotoxicity on cortical neurons by activating extrasynaptic GluN2D-containing NMDARs^25^ but can also activate synaptic GluN2A-containing NMDARs, leading to a neuroprotective effect.^19^ Alternatively, the neurotoxic *versus* neuroprotective effects of tPA may reflect different effects of endogenous *versus* exogenous tPA or of chronic *versus* acute treatments. Thus, as previously suggested,^20^ our present data show that tPA may have opposite effects depending on its concentration, with the low concentrations that are protective and the higher concentrations of sc-tPA that are deleterious. In addition, we propose here that tPA may differentially influence neuronal fate and signaling pathways as a function of its conformation with sc-tPA and tc-tPA.

Endogenous tPA is produced and released under its single chain-form (sc-tPA)^26^ and is 90% present under its single-chain form in Alteplase when used for thrombolysis (see Figure 1b, lane no cell). When released, it can be rapidly converted into its two-chain form (tc-tPA) by plasmin^26, 27^ present at the cell surface or in solution. Thus endogenous plasmin may directly influence the sc-tPA/tc-tPA ratio. It is now well admitted that the tPA-mediated potentiation of NMDAR signaling is dependent on its proteolytic activity.^15, 17^ However, both plasmin-dependent and plasmin-independent mechanisms have been reported.^15, 17, 28^ Here we demonstrate that potentiation of NMDAR signaling and subsequent neurotoxicity is a phenomenon restricted to sc-tPA and dependent of proteolytic activity. Whether tPA would require low-density lipoprotein receptor-related protein (LRP) or not in order to enhance NMDAR signals could depend on the type of neurons, their state of maturation or the presence of astrocytes in cultures.^17, 29^

Several *in vitro* studies reported antiapoptotic effects of tPA on neurons^9, 10^ and oligodendrocytes.^22^ In agreement with our data, despite the heterogeneity of the toxic paradigms used, they all show that this trophic function of tPA occurs independently of its proteolytic activity. Two candidates have been proposed as the receptors mediating the antiapoptotic effects of tPA: Annexin II and EGFRs.^10, 22^ For instance, tPA can bind EGFRs on oligodendrocytes through its EGF-like domain, induces phosphorylation of EGFRs and subsequent signaling pathways, leading to antiapoptotic effects. We evidenced here that both sc-tPA and tc-tPA are antiapoptotic in neurons through a mechanism involving a EGFR-dependent pathway.

Accordingly, data from studies in transgenic mice overexpressing tPA in neurons (T4 mice) suggested that tPA can have neuroprotective effects.^19, 20^ In one of these studies, the authors propose a mechanism that is dependent of the activation of NMDARs and independent on plasmin. It is interesting to note that, in our hands, the tPA-dependent activation of EGFRs led to a reduced NMDAR-mediated calcium influx. These data unmask a tPA-dependent crosstalk between NMDARs and EGFRs, with NMDAR and EGFR that can form complexes. Similarly, in PC12 cells (pheochromocytoma cells) tPA was reported to control a crosstalk between NMDARs and Trk receptors, a mechanism that involves LRP1 and that is differentially controlled by the dose of tPA.^30^

In conclusion, our present study provides information that help to understand how tPA can positively or negatively control neuronal fate.

## Materials and Methods

### Experimental procedures

Experiments were carried out in accordance with the European Communities Council Directive (86/609/EEC) and were approved by the local ethical committee.

### Chemicals

NMDA, (+)5-methyl-10,11-dihydro-5H-dibenzo(a,d)cyclopentaen-5,10-imine maleate (MK801) and *N*-(3-Chlorophenyl)-6,7-dimethoxy-4-quinazolinanine hydrochloride (AG1478) were purchased from Tocris (Bristol, United Kingdom). 2,7-Bis-(4-amidinobenzylidene)-cycloheptanone-1-dihydrochloride salt (tPA-stop) was purchased from American Diagnostica (Stamford, CT, USA). Trasylol (aprotinin) was a gift from Bayer HealthCare AG. Iressa (Gefitinib) was a purchased from Selleckchem (Houston, TX, USA). 6-Aminocaproic acid, ascorbic acid, bFGF, Dulbecco's modified Eagle's medium (DMEM), HEPES buffer solution, hydrocortisone, poly-D-lysine, cytosine *β*-D-arabinoside, Arginine, Tween-80, phosphoric acid and 0.4% Trypan Blue Solution, fetal bovine serum and horse serum were from Sigma-Aldrich (L'Isle d'Abeau, France). CNBr-activated Sepharose 4B was from GE Healthcare (Orsay, France). Plasmin was prepared as described.^30^ Laminin, lipid concentrate and penicillin–streptomycin were purchased from Invitrogen (Cergy Pontoise, France). Endothelial cell basal medium (EBM-2) was purchased from Lonza (Levallois, France).

### Sources of tPA

A human recombinant tPA purified from Chinese Hamster Ovary cells (Actilyse, >95% single-chain) was used as single chain tPA (sc-tPA). Two-chain tPA (tc-tPA) was prepared by overnight incubation of sc-tPA with plasmin-coupled Sepharose 4B at 37 °C, followed by a 2 h incubation with immobilized aprotinin to eliminate traces of free plasmin. Both sc-tPA and tc-tPA were dialyzed in a vehicle containing 0.5 M ammonium bicarbonate for neurotoxocity studies or in the Actilyse buffer (arginine, phosphoric acid and Tween-80) for video calcium imaging. Finally, both sc-tPA and tc-tPA were characterized as we previously described.^18^

### Neuronal cell cultures

Culture of cortical neurons were prepared from fetal mice (E14-15) as previously described.^25^ Cytosine *β*-D-arabinoside (10 *μ*M) was added after 3 days *in vitro* (DIV) to inhibit glial proliferation. Various treatments were performed either after 7 DIV for apoptotic paradigms or 12–13 DIV for NMDA-mediated neurotoxicity assays, calcium imaging experiments and immunoblottings.

### tPA and EGFR immunoblottings

tPA immunoblottings were performed using a polyclonal goat antibody (Santa Cruz Biotechnology – sc5239; 1 : 1000, Heidelberg, Germany) followed by incubation with the appropriate peroxidase-conjugated secondary antibody. Phospho EGFR (Tyr 1173), total EGFR and actin immunoblottings were performed using a polyclonal rabbit antibody (Cell Signaling – 4407; 1 : 1000, Leiden, The Netherlands), a polyclonal rabbit antibody (Cell Signaling – 4267; 1 : 1000) and a polyclonal rabbit antibody (Sigma Aldrich – A2066.2 ml; 1 : 1000), respectively, followed by incubation with the appropriate peroxidase-conjugated secondary antibody. Immunoblots were revealed with an enhanced chemoluminescence ECL2 immunoblotting detection system (Fisher Scientific, Illkirch, France) and imaged in an ImageQant LAS 4000 Device (GE Heathcare, Orsay, France).

### Proximity ligation assay

The proximity ligation assay was performed using the Duolink *In Situ* Kit (Olink Bioscience, Uppsala, Sweden) according to the manufacturer's instructions with the following modifications: PLA probe incubation was of 2 h; amplification step was extended to 2 h. Blocking (1 h at room temperature) and primary antibody (overnight at 4 °C) incubations were performed in a 4% bovine serum albumin (Sigma Aldrich) and 0.2% Triton X-100 solution. Rabbit anti-GluN1 (ab17345, Abcam, Cambridge, MA, USA) and rat anti-EGFR (ab231, Abcam) were diluted (1 : 2000 and 1 : 100, respectively) in the blocking solution. The anti-rabbit (+) PLA probe (1 : 5) along with an anti-rat (−) probe (1 : 100) were diluted in the blocking solution. A Goat anti-rat (Jackson ImmunoResearch Inc., Suffok, UK) were used to make a probe anti-rat according to the manufacturer's instructions using the Duolink Probemaker (Olink Bioscience). Slides were mounted in a mounting medium containing DAPI (4′,6-diamidino-2-phenylindole) and 0.1% deparaphenylene-diamine diluted in phosphate-buffered saline and glycerin. The negative control represents the PLA without the primary antibodies.

Ten stack of picture (0.40 *μ*m per section) were taken from 10 different areas of every well with a confocal microscope (Leica SP5, Leica, Nanterre, France). Punctuas were counted manually using a *z* projection of the stack.

### Crossed immunoprecipitation assays

Supernatants from TNT buffer (50 mM Tris-HCl, pH 7.4, 150 mM NaCl and 0.5% Triton X-100)-lysed cultured cortical neurons (12 DIV) (500 *μ*g of total proteins) were incubated overnight at 4 °C with an antibody raised against the C-terminal end of the GluN1 subunit of NMDAR (2 *μ*g, Santa Cruz Biotechnology – sc1467) and then coupled to protein G-sepharose beads as described by the manufacturer (GE Healthcare) for immunoprecipation procedures. Then immunoprecipitated proteins were separated by 7.5% SDS-PAGE, and immunoblots were revealed with either an antibody raised against total EGFRs (Cell Signaling – 4267; 1 : 1000) or an antibody targeting the C-terminal end of the GluN1 subunit of NMDARs (Santa Cruz Biotechnology – sc1467; 1 : 250) by following the procedure described above (see ‘tPA and EGFR immunoblottings' section).

### Calcium video microscopy

Experiments were performed at room temperature on the stage of a Leica DMI6000B inverted microscope (Leica) equipped with a 150W Xenon high stability lamp and a Leica × 40, 1.3 numerical aperture epifluorescence oil immersion objective. Fura-2 ratio images were acquired with a Digital CMOS camera (Hamamatsu, Massy, France; ORCA-Flash2.8 C11440-10C) and digitized (2048*2048) using the Metafluor 6.1 software (Universal Imaging Corporation, Downington, PA, USA). Cell cultures were transferred into a serum-free medium (HBBSS) and loaded with 10 *μ*M fura-2 AM (Invitrogen) for 45 min at 37 °C. Neurons were washed, and NMDA treatment (25 *μ*M for 30 s) was applied using a peristaltic pump as baselines. Prior a second run of NMDA stimulations, neurons were incubated for 45 min with sc-tPA and tc-tPA alone or in the presence of either AG1478 (a blocker of the transphosphorylation of EGFRs) or an antibody targeting the N-terminal end of the GluN1 subunit of NMDA receptor (GluN1 antibody) previously characterized to prevent tPA–NMDAR interaction.^23^ A mean value of potentiation or inhibition was also measured, including all the recorded cells.

### Excitotoxic neuronal death

Excitotoxicity was induced by exposure of cortical neurons to 50 *μ*M NMDA for 1 h or to 10 *μ*M of NMDA for 24 h in serum-free DMEM supplemented with 10 *μ*M of glycine at 12 DIV and performed as previously described.^25, 31^ The relative amount of tPA bound to cells was assessed by western blotting.

### Induction of apoptosis

SD was induced by exposing neuronal cultures (7 DIV) to a serum-free DMEM supplemented with 10 *μ*M of glycine (+MK-801 at 10 *μ*M to prevent secondary exitototxicity) and characterized as previously described.^31, 32^ The percentage of neuronal death was determined as the number of trypan blue-positive neurons after SD compared with the total number of neurons.

### Statistical analysis

For calcium video microscopy with neurons (Figures 1,2), Shapiro test were used followed by Wilcoxon test to compare preincubation and postincubation responsiveness. Significance levels were defined as #*P*<0.0001. In addition, for group comparison, Kruskal–Wallis tests were used, followed by Mann–Whitney *U*-tests as *post-hoc* tests. Significance levels were defined as *P<0.0001. Other statistical analyses were performed by the two-tailed Kruskall–Wallis' test, followed by *post-hoc* comparisons with the two-tailed Mann–Whitney's test.

## Abbreviations

DIV: days *in vitro*
EGF: epidermal growth factor
GluN1: NMDA receptor subunit 1
LRP: low-density lipoprotein receptor-related protein
NMDA: *N*-methyl-D-aspartate
sc-tPA: single-chain tissue-type plasminogen activator
tc-tPA: two-chain tissue-type plasminogen activator.
