# Supplementary material for: Conformations of tissue plasminogen activator (tPA) orchestrate neuronal survival by a crosstalk between EGFR and NMDAR
Source: Cell Death Dis. 2015 Oct 15;6(10):e1924–. doi: 10.1038/cddis.2015.296 (PMC4632317; doi:10.1038/cddis.2015.296)
Supplement: Supplementary Figure Legends [file cddis2015296x1.doc]

**Supplementary Figure 1. The inhibitory effect of sc-tPA at 10 nM is not due to the presence of tc-tPA.** (**a**) After two NMDA stimulations used as baseline, neurons were incubated 45 minutes in the presence of buffer (control, n= 92 cells), sc-tPA at 10 nM (sc-tPA 10nM, n= 98 cells), tc-tPA at 1 nM (tc-tPA 1 nM, n= 98 cells) prior to a second set of NMDA stimulations. Percentages of potentiation or inhibition after incubation are calculated for each cell. (**b**) Percentages of potentiation or inhibition after incubation are calculated for each individual cell and reported as percentages of responsiveness for each group (mean ± SEM; *: p< 0.0001, Kruskal-Wallis test followed by Mann-Whitney test; #: p<0.0001 Wilcoxon test comparison pre- and post-incubation responses).

**Supplementary figure 2.** Gefinitib, an EGFR tyrosine kinase inhibitor, reverses the inhibitory effect of tc-tPA on NMDARs. **(a**) After two NMDA stimulations used as baseline, neurons were incubated 45 minutes in the presence of buffer (control, n= 90 cells), Gefitinib at 5µM (Gefitinib, n= 89 cells), sc-tPA at 300 nM alone or in combination (sc-tPA, n= 93 cells; sc-tPA + Gefitinib, n= 92 cells) prior to a second set of NMDA stimulations. Percentages of potentiation or inhibition after incubation are calculated for each cell. (**b**) Percentages of potentiation or inhibition after incubation are calculated for each individual cell and reported as percentages of responsiveness for each group. (**c**) In the same protocol, neurons were incubated 45 minutes in the presence of buffer (control, n= 109 cells), Gefitinib at 5µM (Gefitinib, n= 95 cells), tc-tPA at 300 nM alone or in combination (tc-tPA, n= 98 cells; sc-tPA + Gefitinib, n= 102 cells) prior to a second set of NMDA stimulations. Percentages of potentiation or inhibition after incubation are calculated for each cell. (**d**) Percentages of potentiation or inhibition after incubation are calculated for each individual cell and reported as percentages of responsiveness for each group (mean ± SEM; *: p< 0.0001, Kruskal-Wallis test followed by Mann-Whitney test; #: p<0.0001 Wilcoxon test comparison pre- and post-incubation responses).
