# Supplementary figures and images for: Conformations of tissue plasminogen activator (tPA) orchestrate neuronal survival by a crosstalk between EGFR and NMDAR
Source: Cell Death Dis. 2015 Oct 15;6(10):e1924–. doi: 10.1038/cddis.2015.296 (PMC4632317; doi:10.1038/cddis.2015.296)

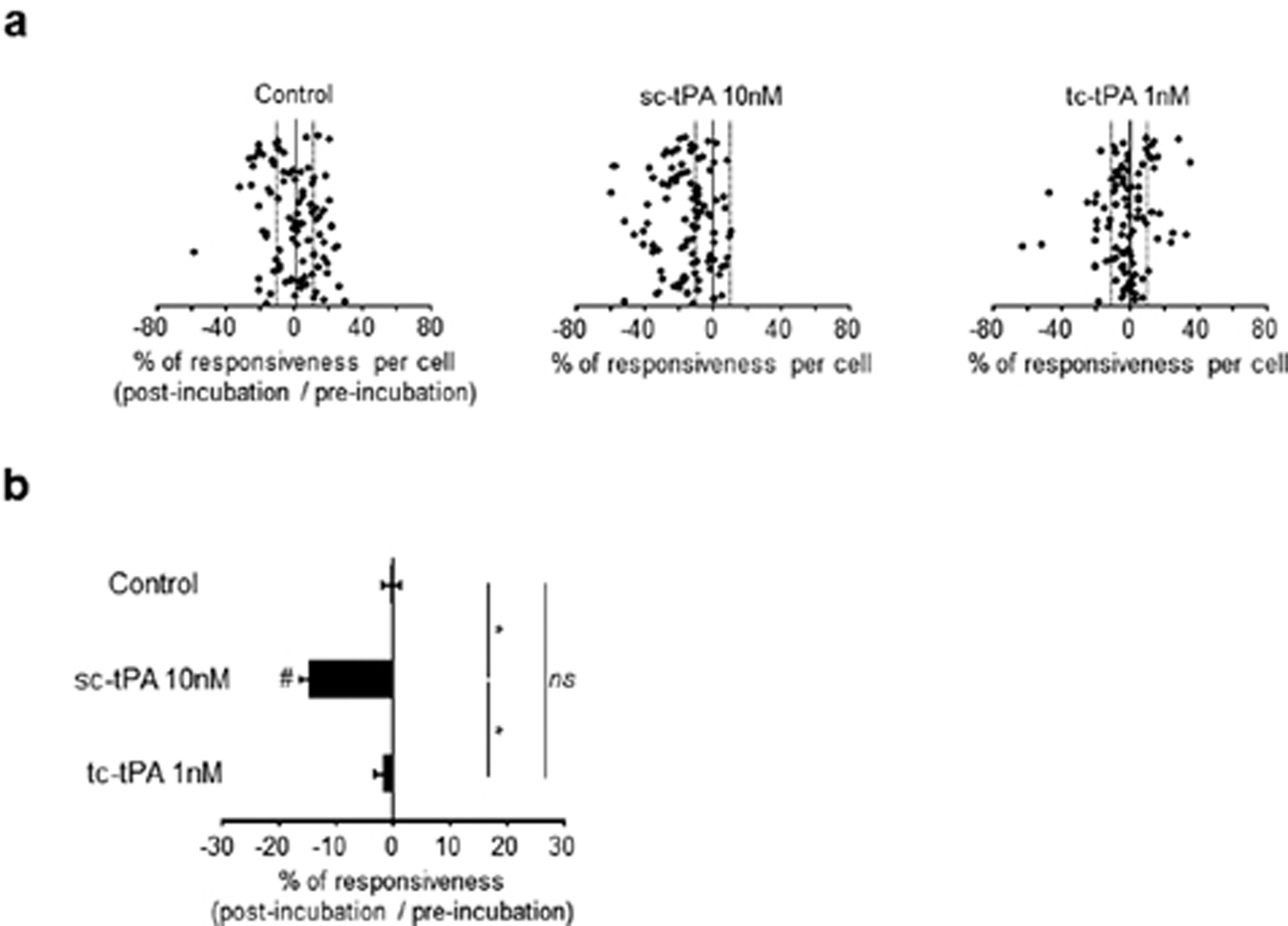

Supplement: Supplementary Figure S1 [file cddis2015296x2.tif]

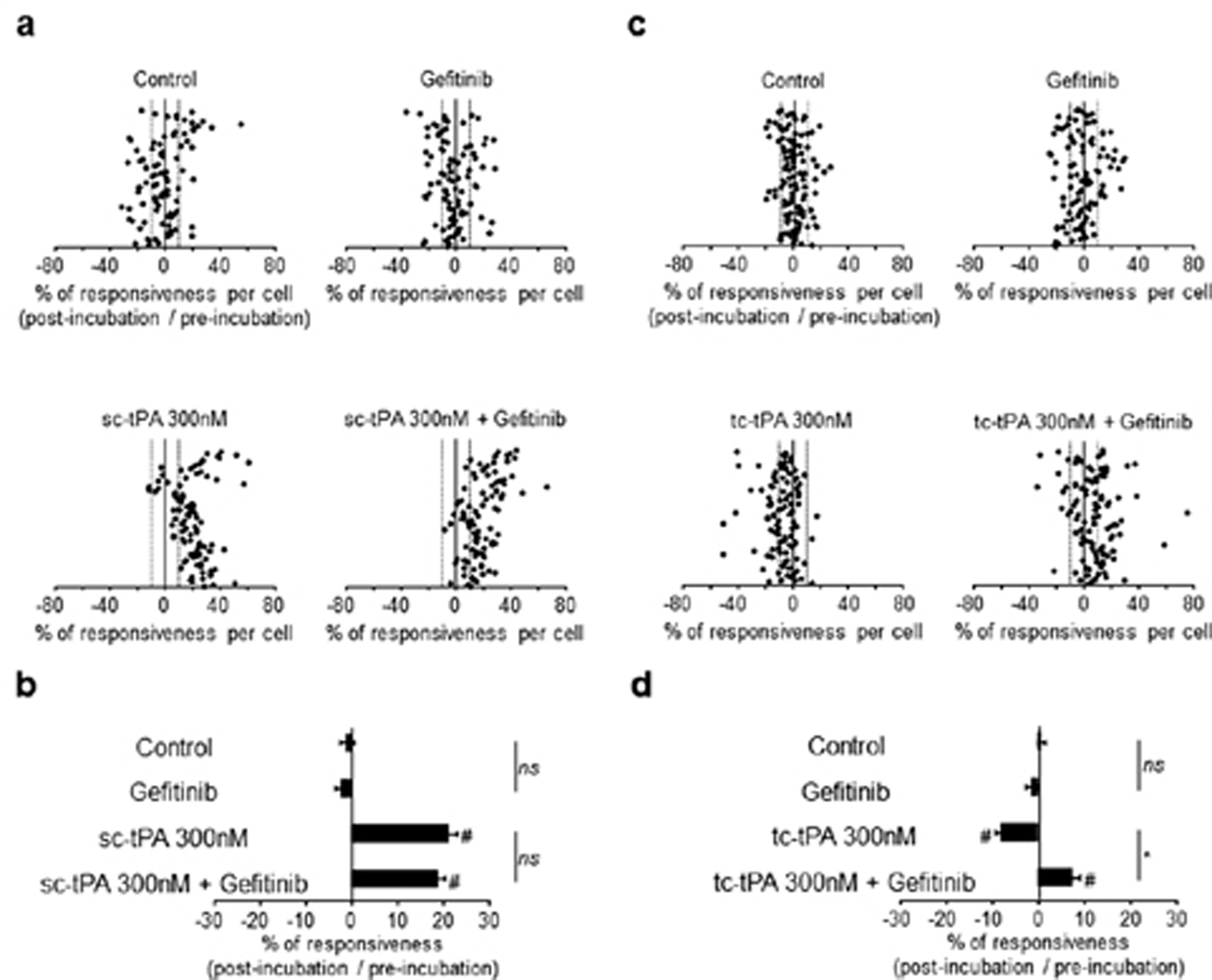

Supplement: Supplementary Figure S2 [file cddis2015296x3.tif]
